# Breast Milk Constituents and the Development of Breast Milk Jaundice in Neonates: A Systematic Review

**DOI:** 10.3390/nu15102261

**Published:** 2023-05-10

**Authors:** Chang Gao, Yixin Guo, Mingxi Huang, Jianrong He, Xiu Qiu

**Affiliations:** 1 Division of Birth Cohort Study, Guangzhou Women and Children’s Medical Center, Guangzhou Medical University, Guangzhou 510623, China; chang.gao@bigcs.org (C.G.); yixin.guo@bigcs.org (Y.G.); mingxi.huang@bigcs.org (M.H.); 2 Department of Women’s Health, Guangdong Provincial Key Clinical Specialty of Women and Child Health, Guangzhou Women and Children’s Medical Center, Guangzhou Medical University, Guangzhou 510623, China; 3 Guangdong Provincial Clinical Research Center for Child Health, Guangzhou Women and Children’s Medical Center, Guangzhou Medical University, Guangzhou 510623, China; 4 Provincial Key Laboratory of Research in Structure Birth Defect Disease and Department of Pediatric Surgery, Guangzhou Women and Children’s Medical Center, Guangzhou Medical University, Guangzhou 510623, China

**Keywords:** breast milk jaundice, breast feeding, neonatal hyperbilirubinemia, free fatty acids, UGT1A1, pregnane-3α,2-β-diol, bilirubin

## Abstract

Breast milk is tailored for optimal growth in all infants; however, in some infants, it is related to a unique phenomenon referred to as breast milk jaundice (BMJ). BMJ is a type of prolonged unconjugated hyperbilirubinemia that is often late onset in otherwise healthy-appearing newborns, and its occurrence might be related to breast milk itself. This review aims to systematically evaluate evidence regarding breast milk composition and the development of BMJ in healthy neonates. PubMed, Scopus and Embase were searched up to 13 February 2023 with key search terms, including neonates, hyperbilirubinemia, and breastfeeding. A total of 678 unique studies were identified and 12 were ultimately included in the systematic review with narrative synthesis. These included studies covered both nutritional compositions (e.g., fats and proteins) and bioactive factors (e.g., enzymes and growth factors) of breast milk and formally assessed the difference in the concentration (or presence) of various endogenous components of breast milk collected from mothers of BMJ infants and healthy infants. The results were inconsistent and inconclusive for most of the substances of interest, and there was only a single study available (e.g., total energy and mineral content, bile salts and cytokines); conflicting or even contradictory results arose when there were two or more studies on the subject matter (e.g., fats and free fatty acids contents and epidermal growth factor). The etiology of BMJ is likely multifactorial, and no single constituent of breast milk could explain all the BMJ cases observed. Further well-designed studies are warranted to investigate the complex interaction between maternal physiology, the breast milk system and infant physiology before this field could be progressed to uncover the etiology of BMJ.

## 1. Introduction

Hyperbilirubinemia, or jaundice, marked by elevated levels of bilirubin in the blood, is a common physiological condition that is displayed in many newborn infants in the early postnatal period [1]. This happens as a result of rapid red blood cell turnover, and its catabolic product and unconjugated bilirubin cannot be effectively eliminated from circulation due to low hepatic capacity and immaturity of the enzyme system in newborns [2]. In most cases, it is a self-limiting condition that resolves gradually within the first postnatal week without the need for specific intervention, unless an exceedingly high level of blood bilirubin is of concern or there are underlying pathological reasons [3].

In 1963, pediatricians Newman and Gross first described a series of prolonged jaundice cases related to the practice of breastfeeding [4]. These neonates had unconjugated hyperbilirubinemia and appeared to be healthy and were ruled out for any pathological concerns. In these infants, interruption of breastfeeding would lead to a significant decline in their serum bilirubin level, which would rise again when breastfeeding was re-introduced. This condition was later named breast milk jaundice (BMJ) [5], and it is often late-onset and affects around one-third of infants [6]. Although the prevalence of neonatal jaundice is reported to be significantly higher among infants of East Asian ancestry when compared to those of Caucasian background [7], regional or racial-specific epidemiological data on BMJ are lacking. Infants with BMJ often have higher peak bilirubin levels and slower resolution [8]. Longer-term and/or higher-dosage exposure to hyperbilirubinemia during neonatal periods has been associated with adverse neurodevelopment both in childhood and adulthood [9,10]. Although acute kernicterus is rarely seen in infants with BMJ [11], it still causes serious concerns and distress for parents. Previous research has shown that a significant proportion of parents believe that breastfeeding cessation is a feasible means to prevent neonatal jaundice in general [12], which might pose a barrier to achieving and maintaining exclusive breastfeeding in this population.

Due to this unique phenomenon that the rise and fall of neonatal serum bilirubin levels are closely related to the interruption and resumption of breast milk [5], it is hypothesized that the development of BMJ is caused by breast milk itself. However, human breast milk contains not only essential nutrients but also living microorganisms, bioactive factors and microRNAs that are tailored for the optimal growth and development of infants [13]. To date, there is no consensus regarding which components of breast milk might trigger the development of BMJ. Identifying the key components of breast milk related to BMJ is crucial for mapping out strategies for managing BMJ while protecting breastfeeding at the same time. Therefore, this systematic review aims to critically evaluate the evidence regarding breast milk composition and the development of BMJ in healthy infants.

## 2. Materials and Methods

This systematic review was conducted following the protocol that has been published in Prospero, an international prospective register of systematic reviews (CRD42023400486). The results in this review were reported according to the Preferred Reporting Items for Systematic Review and Meta-analysis (PRISMA) guidelines, a checklist of which is available in Table S1.

### 2.1. Search Strategy

We searched PubMed, Embase (via Ovid) and Scopus for records up to 13 February 2023 using a combination of the following keywords: breast milk/breastfeeding, jaundice/hyperbilirubinemia and neonates/infants to capture all possible literature. A complete list of the search strategies can be found in Table S2. We restricted all search results to human studies and publications in the English language only, and no other limiting conditions were set.

### 2.2. Inclusion and Exclusion Criteria

There was no restriction regarding the type of study; both interventional and observational trials were considered, but the targeted population was restricted to term infants. Only primary research was included (including short communications with sufficient methodological descriptions). Other types of articles, including case reports, reviews, commentaries and correspondence, were excluded. Studies that reported differences (or presence vs. absence) in any composition of breast milk collected from mothers of BMJ infants and non-jaundiced healthy infants were eligible for inclusion. Studies that focused on physiological jaundice in the early neonatal period, pathological jaundice, or compared breastfed infants with formula-fed infants were excluded. Two authors completed the screening process against the pre-set inclusion and exclusion criteria using the Covidence systematic review software (Veritas Health Innovation, Melbourne, Australia; available at www.covidence.org), and conflicts raised during the process were resolved through consultation with the primary author.

### 2.3. Data Extraction and Quality Appraisal

Using a customized data collection table, two authors performed this process independently to collect study information regarding the author and institutional information, population recruitment and screening procedure, diagnostic criteria for BMJ, timing and tools used for breast milk collection and its subsequent storage condition, and the identified breast milk components that are related to BMJ and relevant detection methods, as well as any statistical analysis performed.

Quality appraisal of individual studies was performed by two independent authors against the Quality Criteria Checklist (QCC) for primary research developed by the American Dietitian Association. The QCC involved ten validity questions assessing the study design, conduct, analysis, and reporting, with four additional questions related to the clinical relevance and significance of the research. The quality of the included studies was rated based on the number of ‘yes’ responses to the validity questions described above, and the final rating system was applied as follows: positive (mostly ‘yes’), neutral (four or more ‘no’), or negative (six or more ‘no’).

### 2.4. Data Synthesis

A narrative synthesis was performed due to the high level of heterogeneity in the identified breast milk composition that is related to the development of BMJ and the methodological approach and reporting units used. Hence, a meta-analysis was not possible, and all included studies were grouped according to the breast milk composition. The statistical significance was retracted from the studies, and where significantly different results were reported, the review authors calculated the relative percentage difference between milk samples from mothers of BMJ infants and healthy infants.

## 3. Results

**Figure 1:**
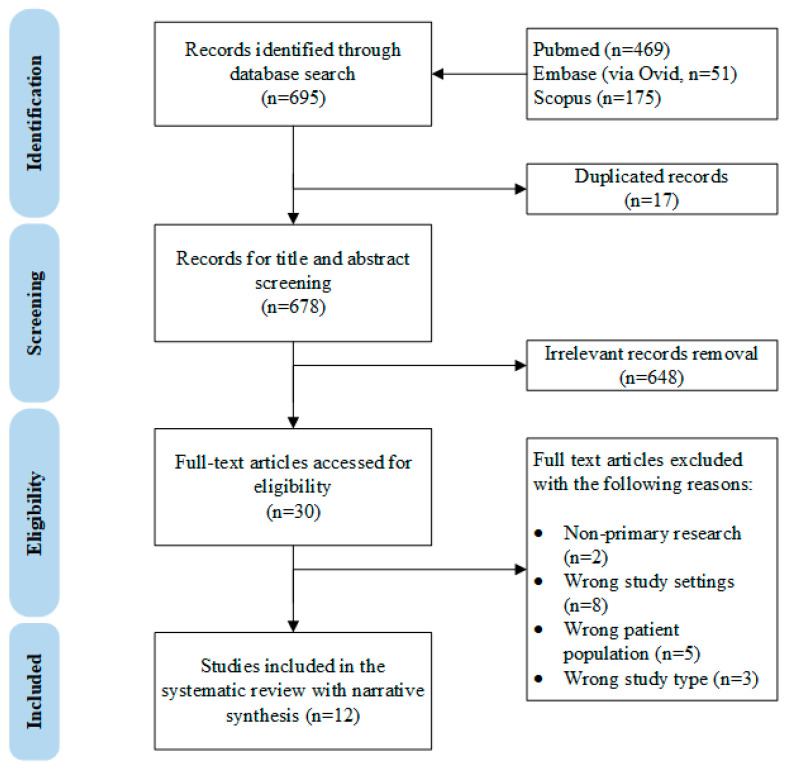
The PRISMA diagram of this systematic review.

The complete study selection process is shown in the PRISMA diagram in Figure 1. Briefly, 695 records were identified through PubMed, Embase (vid Ovid) and Scopus databases. A total of 678 records underwent title and abstract screening after duplicates were removed. A total of 30 records were considered for full-text screening, and 18 were excluded for the following reasons: non-primary research (*n* = 2) [14,15], wrong patient population (*n* = 5, where the studies focused on neonates with physiological jaundice or with congenital anomalies) [16,17,18,19,20], wrong study type (*n* = 3, either case report or in vitro/animal studies) [21,22,23], and wrong study design (*n* = 8, assessed wrong exposure, incorrect comparison groups or lack of formal comparison) [24,25,26,27,28,29,30,31].

A total of 12 unique studies [32,33,34,35,36,37,38,39,40,41,42,43] were included in this review, all of which were observational in nature, and the study characteristics are shown in Table 1. The included studies were dated between 1964 [43] and 2022 [36], with the majority conducted prior to the 2000s [32,34,35,38,39,40]. Nearly half of the included studies were conducted in Turkey (*n* = 6) [33,34,37,38,41,42], and the rest were from six other countries, including China [36], Italy [40], Switzerland [32], the United Kingdom [35], and the United States of America [39,43]. The sample size of these studies ranged from 28 [40] to 139 [39], most of which had a sample size under 100 [32,33,34,35,36,37,38,41,42,43] and followed convenient sampling rather than a pre-determined sample size.

The definition of BMJ in all included studies was prolonged jaundice while the infant appeared to be healthy, and some studies had trialed interruption of breastfeeding [34,35,39] to observe changes in the infants’ serum bilirubin levels for confirmation of BMJ. Most studies have performed extensive laboratory investigations to rule out other potential pathological causes for prolonged jaundice [33,34,35,36,37,38,39,40,41,42,43]. There were minor discrepancies regarding the number/types of additional tests/screening procedures performed, but most studies included common tests, such as the Coombs test, blood typing, G6PD deficiency, etc. The infants’ age at the diagnosis of BMJ varied between 8 [40] days and 42 days [36].

Regarding breast milk sampling, some studies reported the time of the day [32,33,34,35,36,37] and type of milk sampling (fore/hindmilk, or during feeding sample) [33,34,36,37,41], collection method (either manual expression or with aid of pump) [33,35,36,37,38,39,40,41,42,43] and storage condition prior to analysis (either frozen at −20 °C or deep frozen at −80 °C) [33,34,35,36,37,38,39,40,41,42,43].

### 3.1. Energy and Nutritional Composition of Breast Milk

Five studies investigated the differences in energy and nutritional composition (including fats, proteins, lactose, and minerals) in breast milk samples collected from mothers of BMJ infants and from mothers of healthy infants, the pooled results of which are available in Table 2.

Only one single study examined breast milk energy, lactose and mineral content using an automated human milk analyzer and reported no statistically significant differences between the milk of BMJ infants and that of healthy infants [36]. Using the creamatocrit method, Amato and colleagues found that breast milk from mothers of BMJ infants contained significantly higher fat content when compared to that from mothers of healthy infants [32]. In contrast, two other studies using a human milk analyzer [36] and a colorimetric method [39] found no differences between breast milk samples from these two sources. Three studies focused on the free fatty acid concentration in breast milk and measured it using the colorimetric method [39] or selective transmethylation coupled with gas chromatography identification [35]. Although one study [39] found that breast milk collected from mothers of BMJ infants had significantly greater free fatty acid levels when compared to that from mothers of non-jaundiced infants, another study [35] reported that there were no significant differences between these samples. Regarding protein and amino acid concentrations, Poland and co-authors [39] found no differences, and another study [34] reported significantly elevated taurine but not glycine concentrations in milk samples from mothers of BMJ infants when compared to that from mothers of non-jaundiced infants.

### 3.2. Bioactive Components Breast Milk

Ten included studies focused on the bioactive components of breast milk, including enzymes [35,38,39,42], bile salts [35], cytokines [33], epidermal growth factors (EGF) [36,37], steroids [40,43] and antioxidant capacity [41], and how these differed between milk samples from mothers of BMJ and non-jaundiced infants, the results of which are summarized in Table 3.

Three key enzymes related to lipid [35,39] and bilirubin [38,42] metabolism were investigated. Both studies [35,39] agreed that the activity of bile salt-stimulated protein (BSSL) in breast milk was not different between mothers of BMJ infants and healthy infants, but Poland [39] and colleagues found that lipoprotein lipase had higher activity in milk samples compared to mothers of BMJ infants. In addition to BSSL activity, Forsyth et al. also measured the concentration of bile sales cholate and chenodeoxycholate and its relevant ratio and found no differences in the chenodeoxycholate concentration, but elevated cholate concentration and cholate to chenodeoxycholate ratio were found in milk samples collected from mothers of BMJ infants when compared to those collected from mothers of healthy infants. With regard to the breast milk β-glucuronidase concentration, two studies [38,42] with similar sample sizes and methodologies were in agreement that there were no statistically significant differences in milk collected from mothers of BMJ infants in comparison with that from mothers of healthy infants.

Only a single study conducted by Apaydin and colleagues [33] examined various cytokines using a commercial ELIZA kit. The results showed that the concentration of interleukin (IL)-1β was significantly elevated in breast milk samples collected from mothers of BMJ infants compared to those of healthy infants. Nevertheless, the levels of other cytokines, including IL-6, IL-8, IL-10 and tumor necrosis factor (TNF)-α, were similar in both the groups. Two other studies focused on breast milk EGF but reported contradictory results. Guo et al. [36] found a significant reduction of 17%, but Kumral [37] and co-authors found an 80% increase in BMJ milk samples compared to breast milk samples from mothers of non-jaundiced infants. This might be attributed to the differences in the sample size and case-control ratio (1:1 in Kumral et al. [37], 1:2.2 in Guo et al. [36]).

Two other studies [40,43] focused on the steroid pregnane-3α,2-β-diol, and both reported that it could only be detected in breast milk samples from mothers of BMJ infants. Arias [25] and colleagues first used an in vitro model to identify milk with an inhibitory effect on the glucuronidation activity of the liver. The presence of pregnane-3α,2-β-diol found in all BMJ milk samples demonstrated an inhibitory effect, whereas the detection rate was only 77% in BMJ milk samples, as reported by Severi [40] and co-authors.

A single study looked at the antioxidant capacity of milk samples; while the total oxidation status remained stable, the total antioxidant capacity of breast milk samples from mothers of BMJ infants declined and the oxidative stress index was elevated in comparison to that of mothers of non-jaundiced infants, as reported by Uras and colleagues [41].

## 4. Discussion

### 4.1. Summary of Main Findings

This review systematically assessed the currently available evidence regarding the endogenous component(s) of breast milk that is (are) found to be different between mothers of BMJ infants and healthy non-jaundiced infants. This review focused on both the nutritional composition (total energy content, macro- and micronutrients) and bioactive factors (enzymes, cytokines, steroids and antioxidant capacities) of breast milk. For most of these substances of interest (e.g., total energy and mineral content, bile salts and cytokines), there was only a single study available; conflicting or even contradictory results arose when there were two or more studies on the subject matter (e.g., fats and free fatty acids contents, and EGF), which is likely due to the sample size and methodological approach.

### 4.2. Interpretation of the Results

These observational studies arose from the human population and were rooted in some of the evidence generated by in vitro experiments. The unconjugated bilirubin released from red blood cell catabolism is converted to its conjugated bilirubin by the key rate-limiting enzyme UDP-glucuronosyltransferase 1A1 (UGT1A1) in hepatocytes [8]. Research in this area utilized rat liver slices [21], which were incubated with breast milk to observe the inhibitory effect on the glucuronidation process by the liver. This was taken by many as an indication of breast milk endogenous components directly interfering with bilirubin metabolism and causing jaundice. Arias and colleagues were among the first to conduct such research and identified pregnane-3α,2-β-diol as a potential causative agent for BMJ. A subsequent trial was conducted by the same author [25], who observed the changes in serum bilirubin levels in several infants and one adult who had orally administrated pregnane-3α,2-β-diol adjusted according to their body weight. A slight elevation in serum bilirubin was only found in infants of the youngest age (less than 10 days old), but not in older infants (>1 month) and the adult. It seems that this response might be related to the immaturity of the infant rather than solely due to the effect of pregnane-3α,2-β-diol. Later, Severi and colleagues [40] attempted to measure pregnane-3α,2-β-diol in another group of nursing mothers, and most of the BMJ milk samples had detectable levels of pregnane-3α,2-β-diol. However, the authors cross-fed the milk with the highest concentration of pregnane-3α,2-β-diol to a healthy infant between postnatal days 5 and 15, in whom serum bilirubin was not elevated and prolonged jaundice was not developed. This implies that the presence or even a high concentration of pregnane-3α,2-β-diol in breast milk is likely not the primary reason for BMJ.

Similarly, through a series of in vitro experiments, several facts have been established around the role of lipase and free fatty acids in BMJ. The inhibitory effect of breast milk on bilirubin metabolism is exacerbated after prolonged storage, which coincides with the release of free fatty acids due to the presence of lipase [44], and the effect is mediated by chain length and the degree of unsaturation (carbon double bonds) [45]. These long chain (>16 carbon) mono-and polyunsaturated fatty acids were found to be able to suppress the activity of the UGT1A1 enzyme in various substrate types [46], including recombined enzyme system, liver and intestine microsomes with a relatively low concentration compared to breast milk. However, free fatty acids are present in fresh breast milk in trace amounts, the elevation of which is only seen after storage and warming [47], a typical process needed for preparing expressed breast milk for feed. If the free fatty acid concentration is at least partially responsible for the development of BMJ in neonates, it is reasonable to expect that a higher proportion of infants fed with expressed breast milk or donor human milk would develop BMJ compared to those fed directly on the breasts. To date, no such observation has been reported and could potentially be a key area of interest for clinicians for further exploration.

Another major site for bilirubin metabolism is in the intestine, where conjugated bilirubin could be re-admitted to enterohepatic circulation catalyzed by β-glucuronidase. Although two studies [38,42] found that breast milk contained detectable levels of β-glucuronidase, this was not significantly different between milk samples from mothers of BMJ infants and healthy infants. It must be carefully evaluated whether these enzymes would maintain their rigid forms and pose any impact on the reabsorption of bilirubin after going through the digestive tract. Another contributing factor to the elevated enterohepatic circulation of bilirubin is EGF, the concentration of which in breast milk was found to be significantly but very weakly associated with serum EGF and bilirubin level [37]. In addition, the intestinal expression of *UGT1A1* contributes significantly to neonatal bilirubin metabolism. In 2010, a humanized mouse model for studying neonatal hyperbilirubinemia was developed [48], where the authors showed that breast milk feeding suppressed the intestinal expression of *UGT1A1* through the IkB kinase/nuclear factor-kB(IKK/NF-kB) pathway, whereas liver *UGT1A1* expression was still minimal, the combination of which led to the development of BMJ [49]. However, the breast milk samples used in the study were from a single donor only, and no clinical information was available regarding the history of jaundice in the donor’s own children [49]. It is postulated that human milk oligosaccharides (HMO) could block intestinal Toll-like Receptors that further suppress the phosphorylation of IKK and expression of intestinal *UGT1A1* [50]. HMO is the third most abundant substance in breast milk followed by lactose and lipids; the total concentration of it ranges from 11.3 g/L to 17.7 g and decreases gradually with the progression of lactation [51]. Individuals are likely to have a very distinct HMO profile, given that 20% of the population is of non-secretor status and over 200 types of HMO have been identified, and whether a linkage between HMO and BMJ can be established warrants further in-depth research.

Previous research was largely centered around the hypothesis that the development of BMJ is related to breast milk itself. It was not until 2014 that a causal role of UGT1A1*6 genetic mutation on BMJ onset was revealed by Maruo and co-authors [52]. They found that over half of the studied BMJ population carried UGT1A1*6 homozygous mutation, and the other 20% were carriers of heterozygous UGT1A1*6; these infants had significantly higher serum bilirubin levels than those with normal genotype [52]. Altogether, genetic mutation accounts for nearly 70% of BMJ cases in this study, but the underlying causes for the remaining 30% of BMJ infants warrant further research. Recently, several reports have suggested the role of gut microbiota in the development of BMJ in infants. Using 16S rRNA, authors [53,54,55] found that BMJ infants had altered gut microbial composition and metabolic function, and several gut microbiome-derived metabolites were also found to be downregulated in BMJ infants when compared to healthy infants. The essentiality of gut microbiota in bilirubin catabolism was first established using germ-free [56] and antibiotic-treated animal models [57], and several microbial species from the *Clostridium* family were able to hydrolyze bilirubin in vitro [58]. Whether the depletion of these microbes or relevant gut microbial dysbiosis can be linked to the development of BMJ requires further study.

### 4.3. Strengths and Limitations

This review is limited by the fact that a high level of data heterogeneity was found among the included studies, which was largely attributed to the various compounds of breast milk being reported (eight different nutritional components and 14 bioactive factors). In addition, most studies were conducted well before the standardized reporting guidelines were established, hence lacking some level of detail that is considered a ‘must have’ in today’s standard. Moreover, experts in the field of lactation and breast milk research have been advocating to consider breast milk as a biological system rather than isolated components [59], and the studies included in this review only focused on selective components of breast milk. Failing to recognize the complexity of the breast milk system and the potential interaction between the individual components of breast milk in the context of BMJ might prevent us from further understanding its etiology.

### 4.4. Implications for Clinical Practices and Future Research

There is no specific clinical management for BMJ required unless the total serum bilirubin value is exceedingly high, where phototherapy would then be prescribed. This decision is based on the fact that kernicterus caused by hyperbilirubinemia is rare due to advances in medical care, and bilirubin itself has potent antioxidant properties that might be beneficial [11]. Overall, the risk related to BMJ does not outweigh the benefits associated with breast milk and breastfeeding for both mother and infant dyads. Based on the present study, it seems that body and organ system immaturity, as well as genetic susceptibility, are the main reasons for BMJ. The results of this review do not inform any changes to the current practical guidelines; however, a more rigid study design and reporting, taking into consideration the genetic sensitivity of individuals, is warranted in further research.

Through the quality appraisal of individual studies, we have identified some of the common flaws in these included studies that should be considered and/or addressed in future research. Standardized reporting items regarding BMJ diagnostic procedure are preferred to include information such as bilirubin levels, clinical presentations, laboratory tests and postnatal age at confirmation and resolution. A detailed description regarding breast milk sampling, storage and preparation should be reported. Breast milk content (especially fat concentration) [60] varies significantly between different lactational periods within a day, and before and after feeds. It is necessary to take the natural variation of breast milk content into consideration when interpreting study results, and pooled sampling or longitudinal follow-up with multiple time-point sampling might help to overcome this issue.

## 5. Conclusions

The etiology of BMJ is likely multifactorial with genetic susceptibility and neonatal immaturity being the first-line reasons. Based on the results of this review, no single isolated component of breast milk could explain all the BMJ cases observed. It is crucial to first understand the dynamics of breast milk constituents and their interactions with maternal and infant physiology before this field could proceed to further understand the etiology of BMJ.

## Figures and Tables

**Table 1:** Characteristics of the 12 included studies in this systematic review.

Reference, Year, Country	Study Population Recruitment	Diagnosis of BMJ	Breast Milk Collection and Storage	Breast Milk Component Related to BMJ	Quality Rating ^#^
Amato et al., 1985 [32] Switzerland	Recruitment center: NS Duration: NS Inclusion: term infants >38 weeks, normal birth weight Exclusion: bottle feeding supplemented during first 48 h of life Sample size: 98 (50 BMJ, 48 control)	Definition: Healthy neonates without secondary forms of hyperbilirubinemia, but neonatal jaundice Additional tests/screening to rule out other causes: NS Postnatal age: NS	Timing: morning, exact time NS Collection: NS Storage: NS	Component(s): total fat content (%) Method(s): Creamatocrit Statistical comparison: Chi-square test	(+)
Apaydin et al., 2012 [33] Turkey	Recruitment center: Karaelmas University, Department of Pediatrics Duration: April 2009–April 2010 Inclusion: exclusively breastfed infants Exclusion: potential pathological reasons for BMJ Sample size: 80 (40 BMJ, 40 control)	Definition: prolonged jaundice without identifiable pathological cause Additional tests/screening to rule out other causes: blood group compatibility, Coombs test, G6PD deficiency, hemolytic disease, polycythemia, cephalohematoma, asphyxia, hypothermia, intracranial hemorrhage, perinatal infection, hypothyroidism, urinary infection, maternal diabetes mellitus Postnatal age: 2–4 weeks	Timing: 9 a.m.–12 p.m., 1 h after breastfeeding the infant Collection: hand expression, 5 cc Storage: deep-frozen till analysis	Component(s): cytokines (IL-1β, IL-6, IL-8, IL-10, TNF-α, all in pg/mL) Method(s): ELISA kit Statistical comparison: Student’s *t*-test, Mann–Whitney U test	(+)
Arias et al., 1964 [43] United States of America	Recruitment center: Bronx Municipal Hospital Center or specialist referral Duration: NS Inclusion: NS Exclusion: potential pathological reasons for BMJ Sample size: 78 (7 BMJ, 71 control)	Definition: full-term breastfed infants with severe, prolonged, unexplained jaundice Additional tests/screening to rule out other causes: hematocrit, hemoglobin, erythrocyte and leukocyte counts, peripheral blood morphology, Coombs test, blood grouping, serum cephalin cholesterol flocculation, thymol turbidity, glutamic oxaloacetic acid and pyruvic transaminase activities, concentration of albumin and globulin, serological examinations for syphilis, blood cultures, G6PD deficiency, urinary sediment for cytomegalic disease. Postnatal age: NS	Timing: NS Collection: hand expression or manual pump expression Storage: frozen storage till analysis	Component(s): pregnane-3α,2-β-diol Method(s): (1) in vitro experiment to identify milk samples of inhibitory effect on liver samples; (2) isolate and extract the substance of inhibitory effect based on TLC technique and compared to known standards using infrared spectrometry Statistical comparison: NA	(+)
Demirkol et al., 1994 [34] Turkey	Recruitment center: Department of Obstetrics, Istanbul Faculty of Medicine Duration: July and September 1989 Inclusion: exclusively breastfed neonates Exclusion: birth weight less than 2500 g; any disease or malformation Sample size: 65 (12 BMJ, 53 control)	Definition: exclusively unconjugated hyperbilirubinemia; breastfeeding was interrupted to confirm Additional tests/screening to rule out other causes: blood group compatibility, hemoglobin concentration, reticulocyte count and erythrocyte morphology. Postnatal age: ~7 days	Timing: 9 a.m., foremilk before feeding Collection: NS Storage: deep-frozen till analysis	Component(s): taurine and glycine (µmol/dL) Method(s): automatic amino analyzer Statistical comparison: Mann–Whitney U test	(+)
Forsyth et al., 1990 [35] United Kingdom	Recruitment center: NS Duration: NS Inclusion: NS Exclusion: potential pathological reasons for BMJ Sample size: 54 (12 BMJ, 42 control)	Definition: prolonged unconjugated hyperbilirubinemia at 10 days of age, breastfeeding was interrupted to confirm. Additional tests/screening to rule out other causes: liver function and thyroid function infection, blood group analysis. Postnatal age: 10 days	Timing: mid-morning Collection: breast pump Storage: deep-frozen till analysis	Component(s): bile salt (µmol/L), BSSL activity (µmol FFA/mL/min), FFA (mmol/L) Method(s): liquid–liquid extraction then radioimmunoassay; rate of FFA formation; selective transmethylation Statistical comparison: rank correlation test	(+)
Guo et al., 2022 [36] China	Recruitment center: Peking University People’s Hospital Duration: October 2020 and July 2021 Inclusion: Healthy term infants breastfeeding (exclusively or predominantly >70%) Exclusion: preterm infants gestational age <37 weeks Sample size: 94 (29 BMJ, 65 control)	Definition: late onset (occurred after 1st postnatal week), peaked at 2~3 weeks after birth, breastfeeding was interrupted to confirm. Additional tests/screening to rule out other causes: blood group compatibility, Coombs test, G6PD deficiency, hemolytic disease, reticulocytosis, abnormal blood smear, erythrocytosis, cephalohematoma, history of asphyxia, hypothermia, intracranial hemorrhage, cholestasis; mothers had the following conditions: severe liver or kidney disease, with psychological disorders, AIDs, hepatitis B or other infectious diseases. Postnatal age: 42 days	Timing: 9 a.m.–12 p.m., mid-breastfeeding Collection: hand expression or manual pump Storage: deep-frozen till analysis, 5 mL	Component(s): human milk composition (g or kcal/dL); epidermal growth factor (ng/mL) Method(s): automatic human milk analyzer; ELISA kit Statistical comparison: Chi-square test	(+)
Ince et al., 1995 [38] Turkey	Recruitment center: NS Duration: NS Inclusion: exclusively breastfeeding infants Exclusion: NS Sample size: 45 (25 BMJ, 20 control)	Definition: Prolonged jaundice (>1 week) Additional tests/screening to rule out other causes: maternal diabetes, asphyxia, septicemia, cephalhematoma, bruising, blood group incompatibility, hypothyroidism, cholestatic disorder, G6PD deficiency. Postnatal age: 8–30 days	Timing: NS Collection: manual pump Storage: frozen till analysis	Component(s): β-glucuronidase(units/mL) Method(s): ELISA kit Statistical comparison: Student’s *t*-test/Chi-square	(+)
Kumral et al., 2009 [37] Turkey	Recruitment center: Dokuz Eylul University Hospital Duration: January 2007 and June 2008 Inclusion: exclusively breastfed infants Exclusion: potential pathological reasons for BMJ Sample size: 60 (30 BMJ, 30 control)	Definition: late onset at 5–7 postnatal days, peaks around 10th postnatal days Additional tests/screening to rule out other causes: infants with known risk factors, blood group, Coombs test, G6PD, hemolytic disease, reticulocytosis, abnormality of blood smear, maternal diabetes, polycythemia, cephalohematoma, asphyxia, hypothermia, intracranial hemorrhage, perinatal infection, dehydration Postnatal age: 3–4 weeks	Timing: 8–9 a.m., after feeding Collection: manual pump, 5 mL Storage: deep-frozen storage	Component(s): epidermal growth factor (pg/dL) Method(s): ELISA kit Statistical comparison: Mann–Whitney U test; linear regression	(+)
Poland et al., 1980 [39] United States of America	Recruitment center: Hutzel Hospital of Detroit; specialist referral Duration: NS Inclusion: NS Exclusion: NS Sample size: 139 (9 BMJ, 130 control)	Definition: Serum unconjugated bilirubin >10 mg/dL after 7 postnatal days, breastfeeding was interrupted to confirm Additional tests/screening to rule out other causes: standard laboratory investigation performed, but details cannot be retracted. Postnatal age: NS	Timing: NS Collection: manual expression Storage: frozen storage till analysis	Component(s): free fatty acids; lipase; total fat content; total protein content Method(s): (1) in vitro experiment to identify milk samples of inhibitory effect on liver samples; (2) milk samples with inhibitory/non-inhibitory effect were assayed for the above-mentioned milk components with colorimetric method, Statistical comparison: Student’s *t*-test	(+)
Severi et al., 1970 [40] Italy	Recruitment center: NS Duration: NS Inclusion: NS Exclusion: potential pathological reasons for BMJ Sample size: 28 (13 BMJ, 15 control)	Definition: prolonged jaundice, low conjugated bilirubin level Additional tests/screening to rule out other causes: blood group, Coombs test, hemoglobin, erythrocyte and leucocyte counts, peripheral blood morphology, G6PD deficiency Postnatal age: 8–60 days	Timing: NS Collection: manual pump Storage: frozen storage till analysis	Component(s):pregnane-3α,2-β-diol (µg/dL) Method(s):TLC Statistical comparison: NA	(+)
Uras et al., 2010 [41] Turkey	Recruitment center: NS Duration: NS Inclusion: healthy exclusively breastfed newborns Exclusion: severe congenital malformation Sample size: 72 (35 BMJ, 37 control)	Definition: Prolonged jaundice last more than 2 weeks Additional tests/screening to rule out other causes: blood count, peripheral blood smear, Coombs test, liver and thyroid function, urine culture, G6PD, maternal diabetes, birth asphyxia, sepsis, enclosed hemorrhage, hemolytic type hyperbilirubinemia due to blood group incompatibility, other sign or symptoms suggestive of serious illness; maternal hypertensive, renal, hepatic, or hematological disease, taking any medication or tobacco user. Postnatal age: 11–25 days	Timing: NS, foremilk Collection: manual pump, 3 mL Storage: deep-frozen storage till analysis	Component(s): Total antioxidant capacity/status, oxidative stress (mmol Trolox equivalent/L, μmol H_2_O_2_/L, arbitrary unit) Method(s): colorimetric method Statistical comparison: Student’s *t*-test	(+)
Yigit et al., 2001 [42] Turkey	Recruitment center: NS Duration: NS Inclusion: exclusively breastfed infants Exclusion: potential pathological reasons for BMJ Sample size: 40 (22 BMJ, 18 control)	Definition: serum total bilirubin >7 mg/dL on 15th postnatal day Additional tests/screening to rule out other causes: blood group incompatibility, hypothyroidism, cholestatic disorders, asphyxia, septicemia, cephalhematoma, bruising, G6PD deficiency, mothers with diabetes Postnatal age: 15 days	Timing: NS Collection: manual pump Storage: frozen till analysis	Component(s):β-glucuronidase (units/mL) Method(s): ELISA kit Statistical comparison: Mann–Whitney U test	(+)

^#^ Quality appraisal of individual study followed the Quality Criteria Checklist for Primary Research (American Dietitian Association), where the symbols represent the quality rating (+) positive. Abbreviations: BMJ: breast milk jaundice; BSSL: bile-salt stimulated lipase; FFA: free fatty acids; IL: interleukin; NA: not applicable; NS: non-specified; TLC: thin-layer chromatography; TNF: tumor necrosis factor.

**Table 2:** Main results of the included studies regarding differences in the nutritional composition of breast milk samples collected from mothers of BMJ infants and healthy non-jaundiced infants.

**Substances/Measures**	**Sample Size**		Direction of Change or Presence of Substance(s) *	**Reference**
	**Case**	**Comparison**		
**Energy**				
Total energy content	29	65	—	[36]
**Minerals**				
Total mineral content	29	65	—	[36]
**Carbohydrate**				
Lactose	29	65	—	[36]
**Fats and fatty acids**				
Total fat content	50	48	↑39%	[32]
	29	65	—	[36]
	9	130	—	[39]
Free fatty acids concentration	9	130	↑132%	[39]
	12	42	—	[35]
**Proteins and amino acids**				
Total protein content	9	130	—	[39]
Taurine concentration	12	53	↑65%	[34]
Glycine concentration			—	

* Breast milk samples collected from mothers of non-jaundiced infants as controls. “—” represents no statistically significant differences reported,”↑” indicates significant elevation in milk samples from mothers of BMJ infants.

**Table 3:** Main results of the included study regarding differences in the bioactive components of breast milk samples collected from mothers of BMJ infants and healthy non-jaundiced infants.

**Substances** **/Measures**	**Sample Size**		Direction of Change or Presence of Substance(s) *	**Reference**
	**Case**	**Comparison**		
**Enzymes**				
BSSL activity	12	42	—	[35]
	9	130	—	[39]
Lipoprotein lipase	9	130	↑98%	[39]
β-glucuronidase	25	20	—	[38]
	22	18	—	[42]
**Bile salts**				
Cholate	12	42	↑17%	[35]
Chenodeoxycholate			—	
Cholate/Chenodeoxycholate ratio			↑75%	
**Growth factors and cytokines**				
IL-1β	40	40	↑61%	[33]
IL-6			—	
IL-8			—	
IL-10			—	
TNF-α			—	
Epidermal Growth factor	29	65	↓16.8%	[36]
	30	30	↑80%	[37]
**Steroid**				
pregnane-3α,2-β-diol	7	17	100% detection	[43]
	13	15	77% detection	[40]
**Antioxidant feature**				
Total antioxidant capacity	35	37	↓29%	[41]
Total oxidation status			—	
Oxidative stress index			↑35%	

* Breast milk samples collected from mothers of non-jaundiced infants as control. Abbreviations: BSSL: bile salt stimulated lipase; IL: interleukin; TNF: tumor necrosis factor. “—” represents no statistically significant differences reported, ”↑” indicates significant elevation and “↓” represents significant reduction in milk samples from mothers of BMJ infants.

## Data Availability

Not applicable.

## Supplementary Materials

The following supporting information can be downloaded at: https://www.mdpi.com/article/10.3390/nu15102261/s1, Table S1: PRISMA checklist of this review; Table S2: complete list of search strategies used for this review.
