# Supplementary material for: Breast Milk Constituents and the Development of Breast Milk Jaundice in Neonates: A Systematic Review
Source: Nutrients. 2023 May 10;15(10):2261. doi: 10.3390/nu15102261 (PMC10224501; doi:10.3390/nu15102261)
Supplement: Supplementary file 1 [file nutrients-15-02261-s001.zip › Table S2_search strategies.pdf]

**Database**	**Search terms**	**Records (as of 13th Feb 2023)**
Pubmed	(("human milk"[Text Word] OR "milk, human"[MeSH Terms] OR "breast feeding"[MeSH Terms] OR "breast milk"[Text Word] OR "exclusive breastfeeding"[Text Word]) AND (("jaundice"[MeSH Terms] OR "hyperbilirubinemia"[MeSH Terms]) AND ("infant"[MeSH Terms] OR "neonates"[Text Word] OR "infant"[Text Word]))) AND ("humans"[MeSH Terms] AND "English"[Language])	469
Embase via Ovid	Embase <1974 to 2023 February 10><br><br>1 (breast milk or breast feeding).tw. 33231<br>2 (hyperbilirubinemia or jaundice).tw. 56143<br>3 (neonate or infant).tw. 253001<br>4 1 and 2 and 3 149<br>5 limit 4 to (english language and "remove medline records") 51	51
Scopus	TITLE-ABS-KEY ("breast milk" OR "breastfeeding" AND TITLE-ABS-KEY("jaundice" OR "hyperbilirubinemia") AND TITLE-ABS-KEY ("infant" OR "neonate") AND NOT INDEX (medline) AND (LIMIT-TO (LANGUAGE,"English"))	175
